# Febrile illness diagnostics and the malaria-industrial complex: a socio-environmental perspective

**DOI:** 10.1186/s12879-016-2025-x

**Published:** 2016-11-17

**Authors:** Justin Stoler, Gordon A. Awandare

**Affiliations:** 1 Department of Geography and Regional Studies, University of Miami, Coral Gables, FL USA; 2 Department of Public Health Sciences, Miller School of Medicine, University of Miami, Miami, FL USA; 3 Abess Center for Ecosystem Science and Policy, University of Miami, Coral Gables, FL USA; 4 West African Centre for Cell Biology of Infectious Pathogens, University of Ghana, Legon, Ghana; 5 Department of Biochemistry, Cell and Molecular Biology, University of Ghana, Legon, Ghana

**Keywords:** Fever, Malaria, Communicable disease, Diagnostics, Africa

## Abstract

**Background:**

Global prioritization of single-disease eradication programs over improvements to basic diagnostic capacity in the Global South have left the world unprepared for epidemics of chikungunya, Ebola, Zika, and whatever lies on the horizon. The medical establishment is slowly realizing that in many parts of sub-Saharan Africa (SSA), particularly urban areas, up to a third of patients suffering from acute fever do not receive a correct diagnosis of their infection.

**Main body:**

Malaria is the most common diagnosis for febrile patients in low-resource health care settings, and malaria misdiagnosis has soared due to the institutionalization of malaria as the primary febrile illness of SSA by international development organizations and national malaria control programs. This has inadvertently created a “malaria-industrial complex” and historically obstructed our complete understanding of the continent’s complex communicable disease epidemiology, which is currently dominated by a mélange of undiagnosed febrile illnesses. We synthesize interdisciplinary literature from Ghana to highlight the complexity of communicable disease care in SSA from biomedical, social, and environmental perspectives, and suggest a way forward.

**Conclusion:**

A socio-environmental approach to acute febrile illness etiology, diagnostics, and management would lead to substantial health gains in Africa, including more efficient malaria control. Such an approach would also improve global preparedness for future epidemics of emerging pathogens such as chikungunya, Ebola, and Zika, all of which originated in SSA with limited baseline understanding of their epidemiology despite clinical recognition of these viruses for many decades. Impending ACT resistance, new vaccine delays, and climate change all beckon our attention to proper diagnosis of fevers in order to maximize limited health care resources.

## Background

Global funding for malaria alone reached US$2.7 billion in 2013, a threefold increase from 2005 [1], amid calls by leading malaria research institutions to further scale up funding of malaria control programs [2, 3]. The World Health Organization (WHO) estimated that investments would have to grow to US$6.4 billion by 2020, and US$8.7 billion by 2030 to achieve its global target for a 90% reduction in malaria transmission [4]. Amid vast diagnostic resource constraints in sub-Saharan Africa (SSA), malaria has traditionally been the *de facto* presumptive diagnosis for patients experiencing acute febrile illness (AFI), a category that encompasses half of clinical visits in many SSA nations. Successful malaria control efforts have lowered malaria transmission in SSA [5] to the point where bacterial and viral pathogens now drive AFI in the region [6–9]. But the global burden of AFI remains unknown due to limited focus on fever, and estimating this burden is ridden with challenges [10].

While malaria’s landscape ecology has traditionally been rural, the typical SSA capital has experienced persistent annual urban growth rates of 5–6% driven largely by rural-to-urban migration, with some cities growing in excess of 10% annually, implying population doubling every decade [11]. The urban ecology that is home to an increasing proportion of SSA’s population—over half in many nations—supports much lower levels of malaria transmission [12]. Misdiagnosis issues and urbanization trends are conspicuously ignored in high-profile malaria analyses which: purport a larger burden than previously estimated [13]; attribute the averting of 663 million clinical cases solely to insecticide-treated bed nets (ITNs), indoor residual spraying (IRS), and artemisinin-based combination therapy (ACT) [14]; and model optimal deployment of these intervention strategies [15]. In resource-constrained SSA, a region on pace for the world’s highest rates of population growth over the next half-century [16], most febrile illnesses are still presumptively treated as malaria despite growing evidence that, especially in urban contexts, malaria is probably not a major problem. Yet the global health and development communities remain disproportionately focused on malaria control, which often results in an outpouring of funds into preventative consumables and management with relatively little measurement and evaluation. Despite having the best of intentions, this creates a cycle of policy and monetary relationships between international donors, local government, and national malaria control programs that we call the “malaria-industrial complex.”

A socio-environmental approach to understanding the etiology of febrile illness integrates biomedical considerations with the scientific, economic, and socio-political realities wrought by society’s increasing detachment from the physical environment [17]. This is similar to the One Health approach, which recognizes the interconnectedness of humans, animals, and the environment, but with a greater emphasis on the social context within which these actors produce effects on health within social-ecological systems [18]. This paper argues that the global health community needs to convene interdisciplinary groups of experts to embrace an integrated approach for AFI, and offers Ghana as a case study to highlight the obstacles to developing a more comprehensive focus on communicable disease diagnostics across SSA. Malaria is certainly still an important problem in many parts of the world, but malaria control strategies could be far more targeted and efficient if we had a better handle on febrile illness etiology. In the meantime, West Africa—given its growth rate and depletion in capacity following the Ebola epidemic—will continue to be a frontline for a host of neglected tropical diseases that continue to be misdiagnosed as malaria and remain untreated.

## Main body

### Malaria misdiagnosis

Malaria misdiagnosis receives little attention but remains a common problem throughout SSA [19–28], with a greater burden on poor communities [29, 30]. In high-transmission, low-resource settings, health care facilities have historically depended on clinical algorithms for malaria case management [21]. A clinical algorithm will indicate a diagnosis of malaria given a defined set of predictor symptoms and signs such as intermittent fever, chills, and an enlarged liver and spleen [31]. But because the symptoms of non-severe malaria resemble those of most AFI, clinical algorithms for malaria typically result in fewer true-negatives and a higher rate of false-positives [25, 31], thus impeding differential diagnosis [32, 33]. Because the presence of fever is often presumptively treated as malaria, even when malaria may not be circulating [28], the true burden of diseases such as typhoid, influenza, and dengue fever are likely understated in SSA [34]. This was a contributing factor in WHO’s 2010 revision to malaria care guidelines to recommend confirmation of all suspected malaria cases by parasitological test [35]. Yet even where evidence-based guidelines exist for malaria diagnosis, health care provider adherence to guidelines can be problematic [36].

A study from Tanzania highlights the diverse etiology of AFI. Of 870 hospital admissions, 528 (60.7%) patients were clinically diagnosed with malaria, but only 14 (1.6%) actually had malaria parasites upon subsequent blood analysis. Ten different kinds of infections, including bloodstream infections, bacterial and fungal infections, and arboviruses, were all presumptively diagnosed as malaria based on clinical symptoms [19]. But this study was conducted in Moshi, a city of roughly 144,000, which may not be representative of the local disease ecology of large, tropical SSA urban agglomerations like Luanda, Dar es Salaam, Abidjan, Nairobi, or Accra, much less emerging megacities like Lagos or Kinshasa-Brazzaville. We are just beginning to realize how little we know about communicable disease epidemiology in SSA.

Results of studies assessing local rates of malaria misdiagnosis are trickling out of SSA. In Nigeria, 83% of children under 5 years old received ACT even after testing negative for malaria *via* microscopy [37]. Clinical and home-based presumptive treatment of malaria remains common and has probably masked actual malaria transmission reduction in some countries [38], particularly in urban areas. Malaria over-diagnosis in SSA also suffers from high clinical workloads, variable health worker training and lab techniques, and low laboratory capacity [39]. Because many fevers are still treated at home (using both modern and traditional medicines), clinical data greatly underestimates the enormity of AFI in SSA [30, 40]. In hyper- and holoendemic regions, naturally acquired immunity provides protection against malaria, as infected adults routinely maintain a level of parasite prevalence that would otherwise kill a malaria-naïve visitor, but without experiencing overt disease themselves [41]. This further complicates the proper diagnosis of AFI, as febrile patients presumed to have malaria may test positive on a highly-sensitive malaria rapid diagnostic test (RDT) due to a low parasite load that their immune system keeps in check, yet actually be suffering from another infection. We need to rethink the concept of misdiagnosis by systematically establishing the baseline epidemiology for all major sources of fever while also confronting the many non-biomedical perceptions of fever among local communities [42]. The burden of communicable diseases in SSA remains heterogeneous and enigmatic [6], and recent findings raise important questions about the interaction between demography, malaria control, and other neglected tropical diseases. We can look to Ghana as an important case study.

### Ghana: clinical consequences

Ghana’s 2014 population of 27 million was 51% urban [43], though according to WHO, the entire nation is still considered a high transmission zone for *Plasmodium falciparum* malaria, the most dangerous of the malaria parasite species that infects humans [44]. The 2014 World Malaria Report notes over 20,000 malaria deaths in Ghana between 2000 and 2013, with 2,506 in 2013 [1]. More than half of all clinic visits in Ghana are due to febrile illness [45]. Ghana received about US$100 million for malaria control from international and domestic funders in 2013 alone, an amount that fluctuated between $72–110 million from 2010 to 2014 [44]. In 2013 over 75% was spent on insecticides, insecticide-treated bed nets, and antimalarials, while only approximately 13% was spent on diagnostics—an amount less than the amount reportedly spent on management costs—and just 1–2% was spent on monitoring and evaluation [1]. In 2014, the amount spent on diagnostics fell to approximately half of 2013 levels [44]. Many health care facilities still lack RDTs, and just 23% of Ghana’s reported malaria cases were confirmed *via* microscopy or RDT in 2013; the balance were presumptively diagnosed [1].

Ghanaian clinical staff nationwide were found to be diagnosing approximately nearly half of sick children (and nearly 40% of all outpatients) with malaria, with fewer than a third of diagnoses confirmed *via* microscopy [46]. From 2001 to 2006, 47% of outpatient facility visits by children in Accra—and 37% of adult visits—resulted in a clinical malaria diagnosis [47]. The same study also highlighted how rainfall over the prior two months was a significant predictor of malaria morbidity. We have long understood the association between rainfall, mosquito breeding, and malaria [48], and this has contributed toward the institutionalized view that seasonal spikes in fevers during rainy seasons are primarily attributable to malaria. While this is true, rainfall amplifies many other mosquitoes of public health importance such as *Aedes* and *Culex*, as well as water-borne and water-washed pathogens when flood waters breach inadequate sanitation infrastructure [49]. Typhoid fever may be prone to misdiagnosis as malaria during periods of heavy rain, and child pneumonia has been shown to present clinically with malaria-like symptoms in SSA [33, 50]. A 2009–2010 Accra study of 605 feverish children suspected of malaria yielded a parasite-positive rate of just 11% after blood microscopy, yet 80% were diagnosed with malaria and prescribed anti-malarials [51]. All of the evidence suggests that malaria incidence data do not accurately represent Ghana’s malaria burden [46], and are consistent with observations of very low parasitemia levels (1.5%) in Accra made 25 years ago despite similar frequency of fever as in rural areas [52].

Dengue exposure with probable local transmission was recently demonstrated in Ghana [53]. The study tested archived plasma samples from 218 children aged 2–14 years who tested positive for malaria at local health facilities in three Ghanaian cities, and found previous dengue exposure in 21.6% and recent exposure in 3.2% of the children sampled. Notably, this study screened the opposite population—lab-confirmed malaria patients—whom we would not normally evaluate for other sources of infection. Therefore there is a real possibility of dengue infections (and potentially other arbovirus infections) in malaria-negative febrile illness patients, which highlights the complexity of febrile illness in Ghana. These findings are most certainly just the tip of the iceberg for communicable disease epidemiology in Ghana, and across SSA. But clinical revelations must be complemented by advances in environmental and community health.

Insect disease vectors thrive in Ghana’s urban areas—and likewise in most SSA nations—due to poor sanitation infrastructure, regular rainy seasons, widespread household water storage due to limited drinking water availability, and low public awareness of vector control behaviors. Among the most important mosquito vectors are *Aedes aegypti,* the primary vector for dengue, Zika, chikungunya, and yellow fever, and *Culex spp*, vectors for several diseases including West Nile virus, filariasis, and Japanese encephalitis [54–57]. Entomological surveys have documented *Ae. aegypti* in West Africa for over one hundred years [58], and the entomological literature relating to arboviral diseases in Ghana is decades old [59–63]. Dengue fever, for example, does not appear in the Ghanaian medical literature until the 21st century even though dengue was reported in seven West African nations with a mortality rate of about 5% [64], and *Ae. aegypti* has reappeared in mosquito surveys for over fifty years [59, 62, 65–67]. The breeding density and biting behavior of *Ae. aegypti* were found to be sufficient for facilitating an outbreak of dengue in four northern Ghanaian communities over a decade ago, though flaviviruses had still not been isolated from mosquito catches [65]. The last mosquito survey of Accra published in the scientific literature, conducted in 2003, reaffirmed the presence of *Anopheles*, *Culex*, and *Aedes*, but focused on just six communities [66].

*Culex* mosquitoes are the most common mosquitoes of public health importance documented in Accra due to their ability to breed in highly polluted water found in sewers and discharge basins [66, 67]. *Culex* will also breed alongside *Ae. aegypti* in man-made containers and pockets of rainwater that collect in refuse piles. Accra’s historical potable water shortages and subsequent municipal water rationing induce widespread household water storage [68], which in turn exacerbate the breeding of *Aedes* and *Culex* mosquitoes by amplifying the number and density of urban breeding sites. In contrast, Ghana’s primary malaria vector, *Anopheles gambiae*, is a traditionally rural pest which prefers clean, still or slow-moving water near vegetation as is typically found in irrigation canals [60]. *An. gambiae* may be adapting to polluted water in SSA [67, 69–71], but the solid waste problem has never been demonstrated to provide enough *Anopheles* breeding habitat to support malaria transmission relative to the heightened risk for viral infections posed by *Aedes* or *Culex* mosquitoes.

Still, a recent study of health perceptions in Accra affirmed the significance of malaria as a primary health threat that cuts across communities of all socio-economic levels [72]. The linking of malaria to inadequate sanitation was a persistent theme as residents of both high- and low-income neighborhoods associated uncollected refuse and clogged drains with mosquito breeding—and thus malaria—with less concern for traditional sanitation-driven diseases such as typhoid fever. This linkage of malaria and garbage was the most popular health misperception reported across Accra, and likely contributes toward the popular perception of a heavy malaria morbidity burden in Ghana [72]. The general lack of knowledge about malaria transmission among Ghana’s populace is old news, and in some regions, local words for *fever* and *malaria* have historically been used interchangeably [73]. Fever has also not always had such an ubiquitous association with mosquitoes, particularly in rural Ghana [74]. Worse still, previous studies in Ghana have observed that in the absence of an intervention, higher knowledge of malaria is not necessarily associated with protective behaviors [75, 76]. In summary, the accumulated evidence from Ghana beckons an approach to fever that integrates biomedical advances with social, behavioral, and environmental levers of patient management.

### A socio-environmental approach

There are significant gaps in biomedical diagnostic capacity and a staggering lack of baseline data describing the underlying etiology of AFI cases in SSA. Improving differential diagnoses for AFI requires better physician training and clinical algorithms that pinpoint hallmark features [77], and are contextualized by rich local communicable disease epidemiology. The expansion of clinical screening thus suggests the reprioritization of limited health resources.

We need to return to public health basics: community-level surveillance coupled with rigorous assessment of knowledge, attitudes, and practices related to febrile illnesses, an approach that has been used in Ghana in a variety of malaria-related studies [75, 76, 78]. Many rich geographic data sets have been used in previous works on health, poverty, and place in Accra, including georeferenced census data, land cover data derived from satellite imagery, water and other infrastructure availability, and household health data, including self-reported malaria (i.e. a proxy for febrile illness) [79]. Practitioners can leverage prior research on characterization of urban neighborhoods to produce high-resolution risk maps of environmental risk factors such as vector species. Traditional epidemiological methods can link environmental factors, demographic characteristics, health behaviors, and hallmark symptoms with confirmed diagnoses. These are precisely the tools used in outbreak investigations, though many SSA nations still have underfunded surveillance systems that hamper timely outbreak detection and response, particularly in those West African nations whose clinical and public health infrastructures were devastated by Ebola in 2015 [80]. The Integrated Disease Surveillance and Response (IDSR) framework, adopted by the WHO African Regional Office for Africa in 1998, aims to improve detection and response by public health managers and decision-makers in African nations and shows great promise for strengthening surveillance systems. But IDSR has not been a panacea, as a recent implementation in northern Ghana has been fraught with “low priority for surveillance, ill-equipped laboratories, rare supervision and missing feedback” [81]. The implementation of the International Health Regulations [IHR(2005)], which became international law in 2007, has also reported gaps in disease surveillance capacity as well as leadership, communication, and collaborative challenges [82], though it remains an important vehicle for responding to global public health emergencies [83].

But we also need to improve our understanding of how patients and clinicians perceive AFI, craft targeted educational messages—which integrate our vector ecology findings—for both the public and clinicians, integrate new diagnostic heuristics into clinical practice, and measure the heuristic’s effectiveness. Many social, economic, and institutional factors are associated with the propensity for seeking care for AFI. Previous work in Tanzania showed that AFI patients did not necessarily pressure clinicians into issuing a malaria diagnosis, and that malaria over-diagnosis may have been attributable to a mismatch between patient expectations and clinician perceptions [22]. Clinicians, on the other hand, were subject to pressures from their initial malaria-centric training, their peers, and perceived patient expectations, in addition to having shared rationales about malaria being easier and more socially acceptable to diagnose [21]. The problem of misdiagnosed AFI in SSA is therefore partly knowledge- and behavior-driven. This requires both patient- and clinician-centric approaches to integrating health education with technology-driven diagnostic changes.

### Technology and training

Many RDTs for tropical infections are new, and some have not yet been rigorously tested in SSA [84]. There remain unknown issues of cross-reactivity among bacterial and viral families, which has remained a problem around the world among flaviviruses [85, 86]. Cross-reactivity has been observed between dengue and both chikungunya and leptospirosis, as well as in some malaria-positive patients [87]. Molecular methods do not have these limitations, but the cost and technical and logistical expertise associated with such methods currently limit rapid deployment and geographically-scalable solutions to differential diagnosis. Commercial serology kits, RDTs, and innovative point-of-care diagnostic products are more suitable, but require ongoing characterization of cross-reactivity as our knowledge of communicable disease epidemiology grows. Fortunately, some new RDT products have demonstrated better sensitivity and specificity than microscopy when compared to DNA-based polymerase chain reaction [88]. Other innovations, such as TaqMan array cards that can simultaneously detect dozens of pathogens at a time in less than three hours, show promise as screening tools during outbreak investigations [89]. New mobile and handheld technology also has the potential to transform diagnostic accuracy. Recent trials of tablet-based diagnostic algorithms have vastly improved AFI diagnosis and pushed antibiotic prescription rates—which are largely driven by anti-malarials—from 84 to 15.4% [90]. Mobile devices show promise for improving the management of childhood fevers [91, 92], particularly with electronic versions of WHO’s Integrated Management of Childhood Illness strategy [93].

Improved health care worker training will be crucial, as prior research has shown that merely the presence of new diagnostic technology such as RDTs in the absence of training can lead to wasted costs, and that the combination of RDTs and training leads to more sustainable improvements in the quality of diagnosis [94]. For example, although 41 of 44 nations in the WHO African Region now require malaria diagnostic testing across age groups and RDT procurement has soared, diagnostic processes and treatment still vary widely [95]. Diagnostic testing in SSA continues to be limited by RDT stockouts, inefficient patient flow in health care facilities, and lack of provider adherence to test results. In many settings, physicians are reluctant to prescribe the treatment that is appropriate given diagnostic results, and additional health education is thus essential for clinicians [45].

In Ghana, active learning approaches that engage clinicians with the medical literature and local research teams have previously been associated with improved clinical practices in malaria care [96]. But efforts to improve the training of Ghanaian health care workers with regard to AFI are subject to a variety of socio-cultural considerations [97]. For example, a recent trial in rural Ghana demonstrated that the introduction of RDTs in absence of microscopy can substantially reduce malaria over-diagnosis and over-prescription of anti-malarials, but that there is limited impact where microscopy already exists [98]. But these modern nuances in care are mediated by historical difficulties deploying trained and motivated staff in all regions of Ghana [99, 100]. Patient perceptions may also influence diagnosis of febrile illness [101]. An evidenced-based approach to febrile illness thus requires a nimble health education component that motivates and empowers health care workers and patients alike to value differential diagnosis of AFI. Greater community member awareness of malaria transmission has been associated with better adherence to vector control interventions [102, 103], and we can leverage these lessons toward proper management of fever. A prototype for febrile-illness-related health communication is currently being piloted as a “Test and Treat” campaign in Nigeria by a group called Support to National Malaria Programme (SuNMaP).

## Conclusion

A socio-environmental approach to acute febrile illness etiology, diagnostics, and management provides several opportunities for integrated research into: (1) scalability of recent advances in diagnostic technology both in the lab and field; (2) geo-demographic and socio-environmental correlates of specific communicable diseases that contribute to SSA’s febrile illness burden; (3) the environmental and human behavioral drivers of vector breeding, as well as assessing integrated vector management techniques that have been successfully deployed in other tropical urban contexts; and (4) the testing of various community-level educational and behavioral interventions related to febrile illness knowledge and care-seeking, particularly given the matrix of caregivers (family, pharmacists, physicians, traditional healers, etc.) available within relatively close proximity in any large sub-Saharan African city. Toward these ends, we summarize some proposed key solutions ranging from clinical to institutional scales:

- Renew the focus on differential diagnosis in tropical medicine and public health training;
- Initiate or improve community-level health communication about febrile illness causes and care;
- Pilot the reallocation of malaria-related health care resources in low-transmission urban settings to support febrile illness diagnostics (which includes malaria);
- Encourage local and international funding agencies to prioritize health worker training and laboratory diagnostic capacity.

As the global health community braces itself for the next Ebola- or Zika-like pandemic and sets new milestones in accordance with the United Nations’ Sustainable Development Goals, we must not lose sight of the basics: Africa’s communicable disease burden—and consequently our world’s—will persist until we commit to getting the diagnosis right the first time. As attention turns to non-communicable diseases in the Global South in recognition of these nations’ double burden of disease [104, 105], it is increasingly clear that we still have much to learn about infectious disease morbidity and mortality in SSA. And that’s to say nothing of emerging ACT resistance creeping across South Asia [106], modest results in malaria vaccine trials [107, 108], and the impact of climate change on communicable disease epidemiology [109], all of which present critical, near-term challenges that underscore the urgency of proper AFI management in SSA. Contemporary neglect of communicable disease epidemiology in SSA is perhaps best captured in comments from Wilber Downs during his Thirty-Sixth Annual Theobald Smith Memorial Lecture at the New York Society of Tropical Medicine in 1974, despite four decades of medical advances:

Whose responsibility is it to engage in such a program? Certainly the local governments have prime responsibility, backed up by the World Health Organization for necessary inter-regional studies. Interested foreign governments, mission groups, and foundations, it is to be hoped, will provide backing, as they have done in the past. But I cannot permit myself to be optimistic. At this time what is being done by all parties combined to improve the situation with respect to the diagnosis of infectious diseases in Africa is utterly inadequate [110].

Misdiagnosis of febrile illness persists as an understudied health problem in SSA, while global focus on malaria eradication has bred a malaria-industrial complex that diverts resources from more common sources of acute fever, especially in urban areas. Rates of malaria misdiagnosis are exacerbated by limited health care resources, clinician and patient perceptions of fever, and limited community knowledge of disease ecology. A socio-environmental approach to febrile illness would help overcome many social and clinical barriers to effective diagnosis and patient management.

## Abbreviations

ACT: Artemisinin-based combination therapy
AFI: Acute febrile illness
IDSR: Integrated Disease Surveillance and Response
IHR(2005): International Health Regulations
IRS: Indoor residual spraying
ITN: Insecticide-treated bed nets
RDT: Rapid diagnostic test
SSA: Sub-Saharan Africa
SuNMaP: Support to National Malaria Programme
WHO: World Health Organization
